# Advances in the Development Ubiquitin-Specific Peptidase (USP) Inhibitors

**DOI:** 10.3390/ijms22094546

**Published:** 2021-04-27

**Authors:** Shiyao Chen, Yunqi Liu, Huchen Zhou

**Affiliations:** School of Pharmacy, Shanghai Jiao Tong University, 800 Dong Chuan Road, Shanghai 200240, China; shiyao_chen@sjtu.edu.cn (S.C.); yqliu93@sjtu.edu.cn (Y.L.)

**Keywords:** ubiquitin-specific peptidases, signaling pathways, drug screening, USP inhibitors

## Abstract

Ubiquitylation and deubiquitylation are reversible protein post-translational modification (PTM) processes involving the regulation of protein degradation under physiological conditions. Loss of balance in this regulatory system can lead to a wide range of diseases, such as cancer and inflammation. As the main members of the deubiquitinases (DUBs) family, ubiquitin-specific peptidases (USPs) are closely related to biological processes through a variety of molecular signaling pathways, including DNA damage repair, p53 and transforming growth factor-β (TGF-β) pathways. Over the past decade, increasing attention has been drawn to USPs as potential targets for the development of therapeutics across diverse therapeutic areas. In this review, we summarize the crucial roles of USPs in different signaling pathways and focus on advances in the development of USP inhibitors, as well as the methods of screening and identifying USP inhibitors.

## 1. Introduction

As a complex regulatory mechanism of biological functions, post-translational modification (PTM) is essential for cell growth and stress response. Generally, intracellular proteins will experience multiple types of modifications after translation, such as phosphorylation, acetylation, methylation, and ubiquitylation, each corresponding to one or more specific functions [1]. Among them, ubiquitylation is responsible for regulating protein–protein interactions, cellular localization, and enzymatic activities of its protein substrates, and it is also related to proteasome-mediated protein degradation. A large number of studies have identified the ubiquitin-driven degradation pathways as one of the most important ways to help maintain protein balance within eukaryotic cells [1,2]. Therefore, the ubiquitylation of proteins plays indispensable regulatory roles in various biological phenomena [2].

Eukaryotic cells are equipped to recognize and degrade proteins by the ubiquitin–proteasome system (UPS). Upon conjugated to chains of ubiquitin, proteins are then directed to the 26S proteasome, a macromolecular protease, and degraded [3]. Ubiquitin is a small peptide (8.5 kDa) consisting of 76 amino acids that is ubiquitous in eukaryotic cells. The peptide sequence is highly conserved and contains seven lysine sites (Lys6, Lys11, Lys27, Lys29, Lys33, Lys48, and Lys63), a glycine site at the C-terminus, and a methionine at the N-terminus (Met1). In general, the poly-ubiquitin chain linked by Lys48 is a degradation marker for proteasome, while the Lys63-linked poly-ubiquitin chain usually works with non-proteasome pathways (such as DNA repair, DNA replication, and cell signal transduction) [4,5,6]. It has been reported that the ubiquitin chains connected to the target protein through Lys6, Lys11, Lys27, Lys29 or Lys33 are also related to proteasome-mediated degradation [7]. In addition, under certain circumstances Lys63-linked ubiquitin chains can also bind and target proteins that need to be degraded by the proteasome [8].

Similar to other PTMs, the ubiquitin modification of protein is a dynamic and reversible process. Ubiquitin modification can be removed by a series of ubiquitin-specific proteases, which is called deubiquitylation. These proteases are named deubiquitinases (DUBs). Deubiquitinases specifically recognize and excise the tumbling molecules on the target protein, and also participate in the editing of poly-ubiquitin, thus playing an important role in the cleavage of ubiquitin precursors and ubiquitin monomers [9,10]. DUBs also regulate gene expression, apoptosis, cell cycle, DNA repair, and cytokines [11,12,13,14,15].

There are nearly a hundred known DUBs including cysteine proteases (USPs, UCHs, MJDs and OTUs) and metalloproteinases (containing metal catalytic domains) according to different catalytic mechanisms. They are divided into the following superfamilies: ubiquitin-specific protease (USP), ubiquitin C-terminal hydrolase (UCH), ovarian tumor protease (OTU), Machado-Josephin domain superfamily (MJD), and zinc-containing metalloproteases [16,17].

In recent years, the vast majority of DUBs have been shown to be associated with a variety of diseases, including cancer, diabetes, neurodegenerative diseases, and infectious diseases [18,19,20,21]. As the largest superfamily with over 50 members, USPs have aroused increasing attention as potential therapeutic targets in recent years [22]. It is interesting to compare USPs with kinases as drug targets since they are both involved in protein posttranslational modifications. Discovery in protein phosphorylation was awarded the Nobel Prize in 1992, and in the past thirty years numerous efforts have been invested in kinase inhibitors which resulted in a good number of clinically approved drugs including Gleevec. However, the research in the ubiquitin system caught the attention of medicinal chemists much later, and only in the past ten years USPs inhibitors have started to gradually emerge. We envision that USPs represent a new reservoir of therapeutic targets, which will reach its prime time in the twenty years to come. To date, no USP inhibitor has yet been approved for clinical use. In this review, we focus on advances in the development of USP inhibitors within the past decade.

## 2. The Ubiquitylation System

### 2.1. The Ubiquitylation and Deubiquitylation Processes

**Figure 1:**
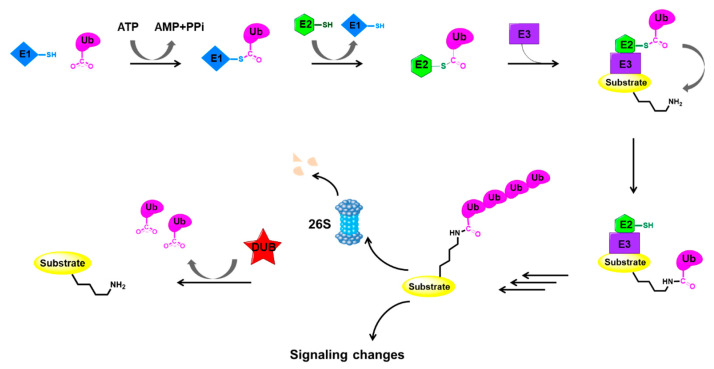
Key events in the ubiquitylation and deubiquitylation process [1,2,3]. E1: ubiquitin-activating enzyme; E2: ubiquitin-conjugating enzyme; E3: ubiquitin-protein ligase; Ub: ubiquitin. Really interesting new gene (RING) E3 ligases which represent the vast majority of E3 ligases are depicted here as an example.

The role of ubiquitin in protein degradation has been discovered and the main enzymatic reactions of this system have been elucidated by biochemical studies. In this system, proteins are targeted for degradation via covalent ligation to ubiquitin, a 76-amino-acid protein. The key biochemical steps in ubiquitylation and deubiquitylation are illustrated in Figure 1.

Ubiquitin-protein ligation requires the sequential action of three enzymes: ubiquitin-activating enzymes (E1s), ubiquitin-conjugating enzymes (E2s), and ubiquitin-protein ligases (E3s) [1,2,3]. Firstly, the E1 enzyme activates the C-terminal carboxyl group of ubiquitin in an ATP-dependent manner, resulting in a covalent high-energy thioester linkage between ubiquitin and the active-site cysteine of E1. Then, the activated ubiquitin is trans-thiolated to an E2, forming a new thioester bond with the E2 catalytic cysteine. Finally, an E3 ligase assists or directly catalyzes the transfer of ubiquitin from E2 to the substrate, generally via the ε-amino group of a substrate lysine. To be specific, E3 ligases from the really interesting new gene (RING) family and RING-related families such as the U-box family can mediate a direct transfer by catalyzing the formation of an isopeptide bond between ubiquitin and the substrate, while the E6AP carboxyl terminus (HECT) E3s and RING-between-RING (RBR) E3s go through a third thioester intermediate with another thioester bond between ubiquitin and the E3 cysteine [23].

Proteins ligated to poly-ubiquitin chains are usually degraded by the 26S proteasome complex that requires ATP hydrolysis. The 26S proteasome is composed of a 20S proteolytic core and 19S regulatory complexes, which is the main proteolytic machine in mammalian cells [24]. The 19S complexes are responsible for the recognition of ubiquitylated proteins, while the 20S core is mainly responsible for catalyzing the degradation of proteins [24].

As mentioned above, the post-translational modification of cellular proteins through ubiquitylation is a dynamic and reversible process coordinated by the action of ubiquitinating and deubiquitinating enzymes. DUBs have fundamental roles in the ubiquitin system through their ability to specifically deconjugate ubiquitin from substrate proteins. Extensive studies suggested that DUBs can act at many different stages throughout the ubiquitin–proteasome pathway [25]. Due to their capability of preventing proteasomal degradation pathways, DUBs regulate the level and/or activities of various proteins such as tumor suppressors, DNA repair proteins, epigenetic modulators, etc., emerging as a compelling target for the development of novel therapies [11,12,13,14,15].

### 2.2. Structural Characteristics of USPs

The USPs, with >50 members, constitute the largest DUBs family described to date. All of them have highly conserved USP domains formed by three sub-domains resembling the palm, thumb and fingers of a right hand. The catalytic site is located between the palm and thumb domains, while the finger domain is responsible for interactions with distal ubiquitin [22]. Furthermore, many USPs exhibit additional domains and terminal extensions, which have important roles in activity and specificity. However, despite their relative structural diversity, most USPs share the common feature of a typical conformational change upon ubiquitin binding, which drives the transition from an inactive form to a catalytically active state. As cysteine proteases, USPs’ catalytic capability mainly depends on the nucleophilic attack by a cysteine in the catalytic site.

Here, we will take USP7 as an example to demonstrate the catalytic process of USPs. As a typical representative of the USPs family, USP7 is also known as herpes virus-associated ubiquitin-specific protease (HAUSP) and is closely related to various diseases such as prostate cancer, colon cancer, lung cancer, and multiple myeloma [26].

**Figure 2:**
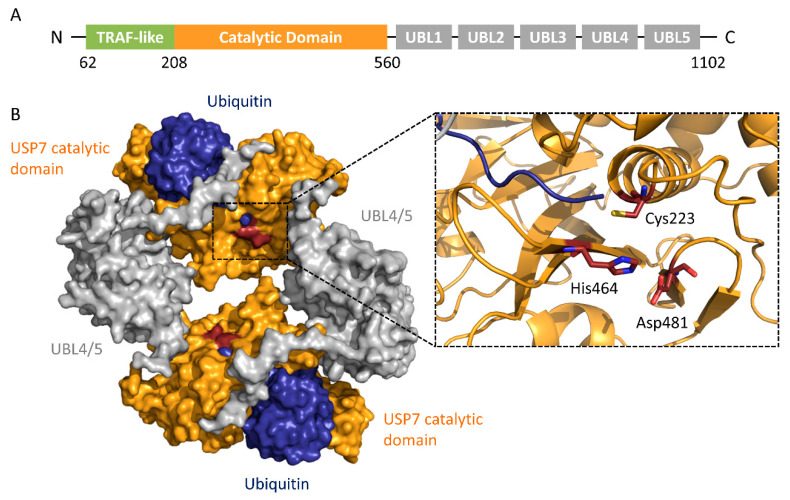
Structure of USP7. (**A**) Schematic of the USP7 domain organization [27]. (**B**) Crystal structure of the dimer of the USP7CD-UBL4/5-ubiquitin complex (PDB ID: 5JTV). Ubiquitin: blue; USP7 catalytic domain: orange; UBL4/5: gray. In this structure, the USP7 N-terminal TRAF-like domain and ubiquitin-like (UBL) domains 1–3 were truncated. The catalytic triad of USP7 (Cys223-His464-Asp481) was shown as red sticks.

USP7 has an N-terminal tumor necrosis factor-receptor associated factor (TRAF) region, a catalytic region, and a C-terminal region (Figure 2). The TRAF region directly binds to substrates such as p53, Mdm2, etc. The catalytic region consists of three domains, resembling the shape of an extended right hand, and forms a binding surface for incoming ubiquitin. The C-terminal region contains five consecutive ubiquitin-like (UBL) regions [27].

The catalytic domain contains the classic catalytic triad: Cys, His, and Asp. The ubiquitin-binding pocket is located in the catalytic region and consists of a papain-like structure and an unfolded finger-like region [28]. The cleavage of the isopeptide bond between the target protein and ubiquitin can be summarized in three steps: binding to the substrate, acylation, and deacylation. First, USP7 specifically binds to its substrate, followed by a conformational change to an activated state. Next, the catalytic cysteine is deprived of a proton by histidine, and the resulting sulfhydryl group undergoes a nucleophilic attack toward the carbonyl Gly76 of ubiquitin from the USP7-ubiquitin intermediate. Finally, the ubiquitin is released by hydrolysis of the thioester bond between USP7 and ubiquitin [29].

## 3. Roles of USPs in Cancers

Aberrant regulation of protein ubiquitylation is closely related to the occurrence and development of tumorigenesis and other pathologies such as neurodegenerative diseases, autoimmunity, inflammatory disorders, infection, muscle dystrophies, etc. [30]. Given that the target proteins for USPs contain a large number of cell homeostasis regulators, as well as products of known oncogenes or tumor suppressor genes, USPs might be attractive and promising targets for the development of novel cancer therapies.

Studies have shown the involvement of USPs in the regulation of multiple known cancer-related pathways, including p53, transforming growth factor-β (TGF-β), protein kinase B (Akt), nuclear factor kappa-light-chain-enhancer of activated B cells (NF-κB), Janus kinase/signal transducers and activators of transcription (JAKs/STATs), and G protein-coupled receptor (GPCR). For example, the overexpression of USP2a stabilizes p53-murine double minute 2 (MDM2) through direct deubiquitylation, without reducing MDM2-mediated p53 ubiquitylation, and thus enhances p53 degradation [31]. Since p53 functions as a tumor suppressor and is vital for normal cellular process controlling, such downregulation of p53 can ultimately cause tumor progression [31]. USP7, however, deubiquitylates both MDM2 and p53, while its affinity to MDM2 is confirmed to be higher [32,33]. Another notable example is USP26. It has been reported to be a novel negative regulator of the TGF-β pathway and the loss of USP26 expression may be an important factor in glioblastoma pathogenesis and breast cancer [34]. Low levels of USP26 degrade drosophila mothers against decapentaplegic protein 7 (SMAD7) and stabilize TGFβ, while high levels of USP26 stabilize SMAD7 by deubiquitylation and form a complex with SMAD ubiquitylation regulatory factor 2 (SMURF2), which degrades the TGF-β receptor by ubiquitylation [34].

Here, in Table 1 we summarized the roles of USPs implicated in tumorigenesis according to the different signaling pathways on which they act.

## 4. Methods in Screening and Identification of Inhibitors for USPs

**Figure 3:**
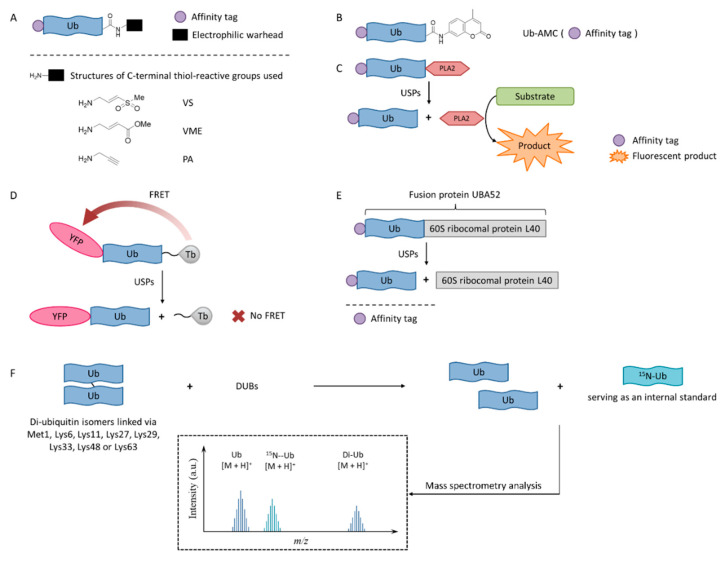
USPs inhibitor screening methods. (**A**) Activity-based probe (ABP) structures; (**B**) Ub-7-amino-4-methylcoumarin (AMC) structure; (**C**) Ub-phospholipase A2 (PLA2) assay; (**D**) Time-resolved fluorescence resonance energy transfer (TR-FRET) assay; (**E**) UBA52 structure and the SDS-PAGE-Coomassie assay; (**F**) Matrix-assisted laser desorption/ionization time-of-flight (MALDI-TOF) method.

In order to enable the continuous discovery and development of inhibitors for USPs, a number of biological testing methods have been developed to screen and identify small molecule inhibitors (Figure 3).

### 4.1. Activity-Based Probes

Activity-based probes (ABPs), in which an electrophilic warhead is introduced onto the C-terminal glycine of ubiquitin, provide a way to test compounds in a cellular environment. Currently reported probe molecules include ubiquitin-vinylmethyl sulfone (Ub-VS) [87], ubiquitin-vinylmethyl ester (Ub-VME) [88], and ubiquitin-propargylic acid (Ub-PA) [89]. They can covalently label the nucleophilic cysteine of DUBs, resulting in a band shift on SDS-PAGE.

The advantage of this method is that it is closely related to the physiological environment of cells. The disadvantage is that it is time-consuming and laborious, so it is not currently recommended for screening USP inhibitors.

Interestingly, in order to overcome the lack of target selectivity, a novel Ub-based activity probe (Rh-M20-PA) bearing specific mutations to achieve selectivity for USP16 was developed by combining structural modelling and computation. A number of USP16-specific inhibitors were successfully discovered using these USP16-selective ABPs [90].

### 4.2. Ub-AMC

Ub-AMC, which has the C-terminus of a ubiquitin molecule linked to 7-amino-4-methylcoumarin (AMC), is a rather simple method, and has been widely applied in the determination of deubiquitinating enzyme activities [91]. The advantages of the Ub-AMC method are low cost per test and commercial availability. However, Ub-AMC is an unnatural substrate, and light-emitting substances (auto-fluorescence or fluorescence quenchers) can interfere with the reading. Later, researchers improved the method by replacing AMC with rhodamine-110 (Rho110) or tetramethylrhodamine, so the wavelength was red-shifted and the interference was reduced [92,93].

### 4.3. Ub-PLA2

In the Ub-phospholipase A2 (PLA2) method, the PLA2 does not directly emit fluorescence after being cleaved from the ubiquitin chain, but it acts on a fluorescent substrate and causes it to emit fluorescence [94]. It is also called the Ub-CHOP method and makes the screening at lower enzyme concentrations possible by amplifying the activity of deubiquitinating enzymes [95]. Its signal intensity and duration are better than the Ub-AMC. Besides, the excitation wavelength is not in the ultraviolet region, and currently there are commercial kits. However, the Ub-PLA2 method is not sensitive to some deubiquitinating enzymes of the UCH family, and its price is higher than the Ub-AMC method. USP inhibitors identified by this method include shionone and P22077 [95].

### 4.4. TR-FRET

The time-resolved fluorescence resonance energy transfer (TR-FRET) method is based on a full-length ubiquitin substrate that is site-specifically labeled with a yellow fluorescent protein (YFP) at the N-terminus and a terbium donor at the C-terminus. This substrate has strong fluorescence resonance energy transfer (FRET) between the two groups, while the cleavage by USPs will decrease the extent of FRET [96,97,98].

The advantage of the TR-FRET method is that it is equally sensitive to the four deubiquitinating enzymes of the UCH family. However, there is no commercial kit available.

The expansion of this application is to use diubiquitin molecules (diUb) as substrates, and this diubiquitin molecule can be connected through different lysine sites to simulate different ubiquitin chain forms [99].

### 4.5. SDS-PAGE-Coomassie

The development of a highly reliable assay based on a readily available SDS-PAGE-Coomassie system using UBA52 as the substrate protein has been reported recently [100]. A number of effective USP2 inhibitors were identified using this assay. Natural substrate UBA52 was used and quantitative measurement was based on the infrared emission of Coomassie dye on SDS-PAGE.

This method uses readily available and inexpensive materials and has excellent reproducibility without the interference problem that is intrinsic to any fluorescence-based approaches. It also has the advantage of using a natural protein substrate, avoiding any artifacts that may be introduced by unnatural substrates. However, this assay was not amenable to high-throughput screening. It is useful for the accurate determination of IC_50_ values during fine-tuning of the structures during the structure-activity studies.

### 4.6. MADAL-TOF

A sensitive and fast assay to quantify in vitro DUBs enzyme activities using matrix-assisted laser desorption/ionization time-of-flight (MALDI-TOF) mass spectrometry has been developed [101]. This method realized the high specificity of many members of the OTU and JAB/MPN/Mov34 metalloenzyme DUB families. It used unmodified substrates, such as di-ubiquitin topoisomers, and can be used to assess the potency and specificity of deubiquitylation inhibitors.

## 5. Recent Development of USP Inhibitors

### 5.1. USP Inhibitors in Clinical Trials

**Figure 4:**
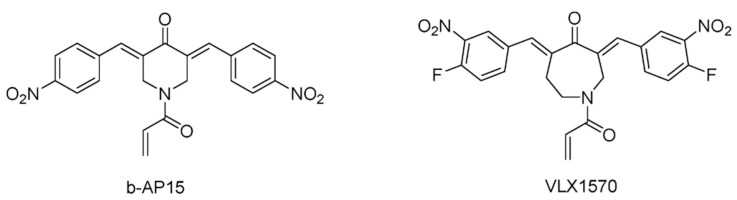
Structures of reported USP inhibitors in clinical trials.

A novel chalcone compound b-AP15 (Figure 4) has been reported to induce the cathepsin-dependent apoptosis by inhibiting the UPS system [102]. Later, it was determined that b-AP15 targeted the proteasome-bound USP14 and UCHL5, which belong to the USP and UCH families, respectively [103]. b-AP15 specifically inhibits the deubiquitylation activity of USP14 and UCHL5, and its affinity to USP14 is slightly higher than that of UCHL5. It was shown that b-AP15 caused the apoptosis of Bcl-2-overexpressing and p53-deficient cells, thus it may serve as a potential treatment for bortezomib-resistant patients [104,105]. b-AP15 was also shown to reduce the viability and proliferation of multiple myeloma cells, which mainly correlates with the reduced levels of cell division cycle 25C (CDC25C), cyclin-dependent kinases 1 (CDK1), cyclin B1 and subsequent caspase-mediated apoptosis and activation of unfolded protein response (UPR) [106]. Besides, the rapid apoptotic response caused by b-AP15 is related to the enhancement of oxidative stress and the rapid activation of the cJun N-terminal kinase and activator protein-1 (JNK-AP1) signaling pathway [107,108]. It also showed efficacy in the multiple myeloma xenograft tumor model. However, the poor solubility and stability of b-AP15 in aqueous solution limited its drugability toward the clinic use.

After an attempted to improve the physicochemical properties of b-AP15, compound VX1570 was obtained in 2015 as the first DUB inhibitor to enter clinical trials [109]. Although there was no significant improvement in the aqueous solubility of VX1570, it required an excipient containing a lower percentage of Kolliphor EL for injection. Compared to b-AP15, it contains an azepane as the central ring instead of a piperidine, swaps the position of nitro groups, and has additional fluoro substitution (Figure 4). It is more inclined to inhibit proteasome DUB activity. VLX1570 binds to USP14 in vitro and inhibits its activity, while its inhibitory activity on UCHL5 is relatively low (Table 2).

In vivo studies on multiple myeloma revealed that VLX1570 was more effective than b-AP15 in inhibiting tumor progression in mice [110].

However, the phase 1 clinical trial of VLX1570 in combination with dexamethasone in patients with relapsed or refractory multiple myeloma was recently suspended due to pulmonary toxicity [111]. In this trial, two patients underwent severe, abrupt, and progressive respiratory insufficiency, which was associated with diffuse pulmonary infiltrates, similar to those rarely noted with bortezomib and other inhibitors of the 20S proteasome. Two possible mechanisms of toxicity have been proposed: the activation of NF-κB and lung inflammation upon drug withdrawal and damage to lung tissues due to the accumulation of active drug metabolites [112].

### 5.2. USP Inhibitors at Early Stage of Drug Discovery

#### 5.2.1. USP1 Inhibitors

**Figure 5:**
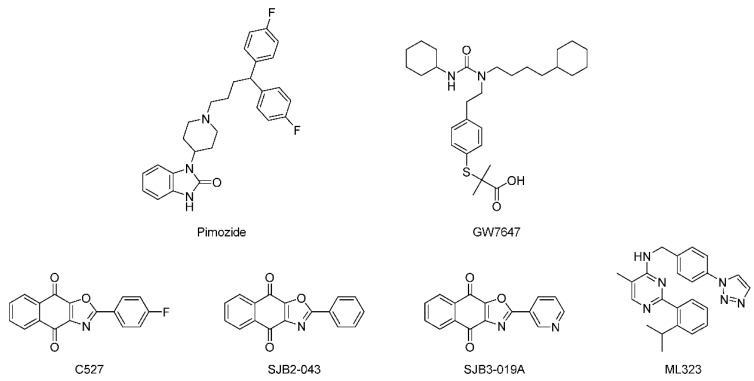
Structures of representative USP1 inhibitors.

Given the fact that USP1 functions as a USP1/UAF1 complex, studies have been conducted to identify inhibitors against the USP1/UAF1 complex. In 2011, the first USP1/UAF1 inhibitors were reported using a Ub-Rho110-based high-throughput screening (HTS) [113]. Among them, pimozide and GW7647 (Figure 5) as the most potent compounds (IC_50_ = 2 and 5 μM) were shown to inhibit USP1/UAF1 by a noncompetitive and reversible mechanism with reasonable selectivity against other human USPs and UCH-family DUBs. Further studies demonstrated that both compounds showed efficacy in cisplatin-resistant non-small cell lung cancer (NSCLC) cells when used in combination with cisplatin, as well as enhanced proliferating cell nuclear antigen (PCNA) and FANCD2 monoubiquitylation in human embryonic kidney 293T (HEK293T) cells. However, pimozide and GW7647 were both known to bind proteins unrelated to DUBs, which has limited their use.

In 2013, C527 (IC_50_ = 0.88 μM) (Figure 5) was identified as a potent USP1/UAF1 inhibitor using a similar Ub-Rho110-based HTS [114]. Later, more potent derivatives SJB2-043 (IC_50_ = 0.544 μM) and SJB3-019A (IC_50_ = 0.0781 μM) were reported. However, their target selectivity remained limited.

To improve the selectivity for USP1/UAF1, following up the previously reported GW7647, ML323 was developed (Figure 5), which was a nanomolar inhibitor of USP1/UAF1 (IC_50_ = 76 nM) with remarkable selectivity over 18 DUBs, deSUMOylase, deneddylase, 70 unrelated proteases, and 451 kinases [115,116]. By inhibiting the deubiquitylation of PCNA and FANCD2, ML323 sensitized cisplatin-resistant NSCLC cells and impaired DNA repair. This study provided ML323 as a best-in-class chemical probe to investigate the function and regulation of the USP1/UAF1 complex.

#### 5.2.2. USP2 Inhibitors

**Figure 6:**
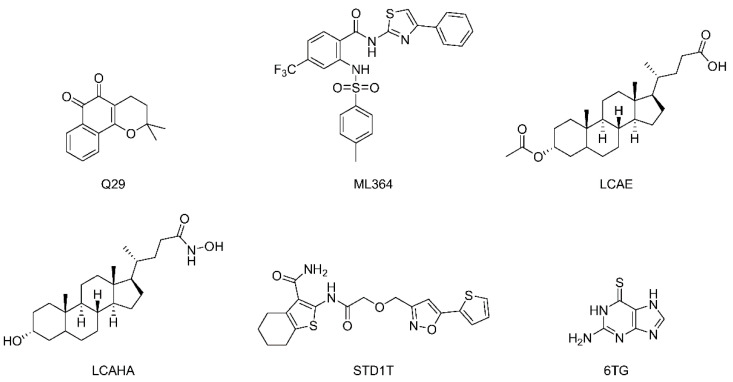
Structures of representative USP2 inhibitors.

By screening a commercial library, a series of ortho quinones with considerable inhibition against USP2 at a concentration of 5 μM was identified [117]. Among them, Q29 (Figure 6) was at that time in advanced clinical trials for pancreatic cancer treatment. Q29 was shown to generate reactive oxygen species (ROS) in the presence of low concentration 1,4-dithiothreitol (DTT), and its activity was due to selective and irreversible oxidation of the catalytic cysteine of USP2.

Through a high-throughput screening followed by structural optimization, ML364 (Figure 6) was identified as a USP2 inhibitor with an IC_50_ of 1.1 μM for the Lys48-linked substrate and 1.7 μM for the Lys63-linked substrate. It was shown that ML364 led to elevated cellular cyclin D1 level and cell cycle arrest in G0/G1 phase, resulting in a downregulation of DNA repair [118].

A series of lithocholic acid (LCA) derivatives was investigated for their anticancer activity, and LCAHA (Figure 6) was found to induce G0/G1 arrest in human colon cancer 116 (HCT116) cells accompanied by decreased expression of cyclin D1, thus leading to an assumption that USP2a was involved [119]. Further studies confirmed that the most potent compound LCAE directly inhibited USP2a activity with an IC_50_ of 5.8 μM by an uncompetitive mode.

A nuclear magnetic resonance (NMR)-based fragment screening led to the discovery of compound STD1T as a selective USP2 inhibitor (IC_50_ = 3.3 μM) (Figure 6) [120].

**Figure 7:**
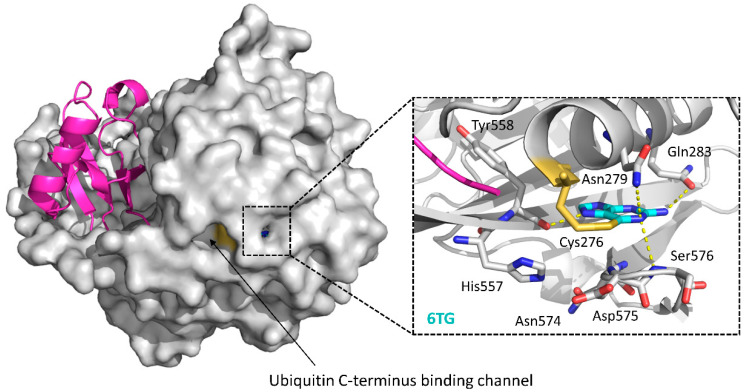
Cocrystal structure of the USP2-Ub-6TG complex and a close-up on the 6TG-binding site (PDB ID: 5XU8). Ubiquitin: magenta; USP2 catalytic domain: gray; 6TG: cyan sticks. In the close-up figure, hydrogen bonds made by 6TG are indicated by yellow dotted lines. Key residues involved in the interactions and the catalytic triad of USP2 (Cys276-His557-Asp574) are shown as sticks, with Cys276 in orange.

Although its inhibitory activity is moderate (IC_50_ = 40 μM), 6-thioguanine (6TG) (Figure 6) was the first and the sole small molecule cocrystal with USP2 to date (USP2-Ub-6TG, PDB ID: 5XU8) [121]. As shown in Figure 7, 6TG has polar interactions with nearby residues Asn279, Gln283, Ser576 and Tyr558, and the sulfur atom of it shows a disulfide bond with the catalytic Cys276. Besides, a 3.2 Å shift of Asp575 toward His557 is also observed [121]. As Asp575 is a conserved residue in the USPs with a role in the protonation of His557 for catalytic competency, the shift of Asp575 may also contribute to the inhibition of USP2.

#### 5.2.3. USP2/4/5/7/8/15/20/28/47/UCHL1/UCHL3/UCHL5 Inhibitors

**Figure 8:**
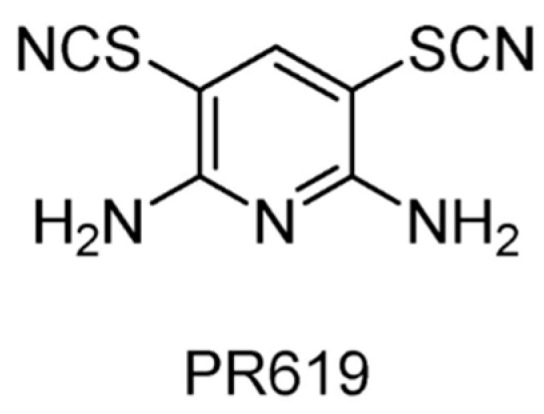
Structure of compound PR619.

Activity-based chemical proteomics was used to screen a diversity-based library for modulators of USP7 activity, and PR619 (Figure 8) was identified as a broad-spectrum DUBs inhibitor (USP2, USP4, USP5, USP7, USP8, USP15, USP20, USP28, USP47, UCHL1, UCHL3, UCHL5), but with limited activity against other families of proteases [122]. In addition, this study demonstrated that DUBs inhibitors induced the accumulation of poly-ubiquitylated proteins in cells without directly affecting proteasome activity. It was also found that PR619 could regulate the microtubule network by inhibiting deubiquitinating enzymes in the improvement of neurodegenerative diseases [123].

#### 5.2.4. USP2/5/8/UCHL1/UCHL3 Inhibitors

**Figure 9:**
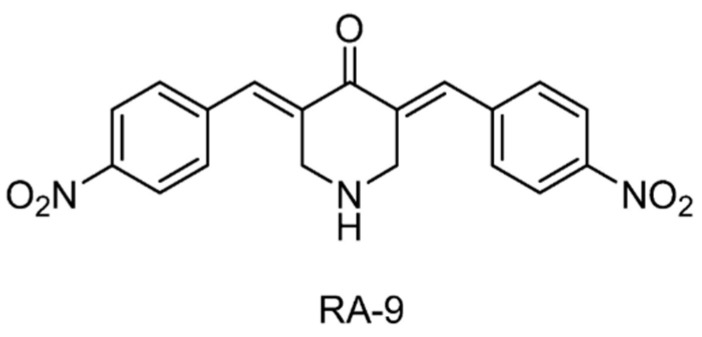
Structure of compound RA-9. The exact chemical structures of AM146 and RA-14 were not reported.

Three chalcone derivatives, AM146, RA-9 and RA-14 (Figure 9), were reported to show direct inhibition of USP2, USP5, USP8, UCHL1, and UCHL3, but with no significant effect on USP7 and USP14. They inhibited tumor cell proliferation by affecting protein ubiquitylation and upregulating p53, p27, and p16 levels [124].

#### 5.2.5. USP2/7 Inhibitors

**Figure 10:**
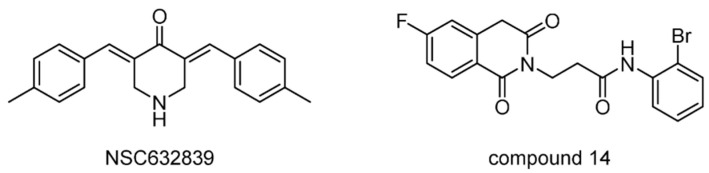
Structures of reported USP2/7 inhibitors.

A previously reported DUB inhibitor, NSC632839 (Figure 10), was identified as the inhibitor of USP2/7. It also exhibited the inhibition of deSUMOylase SUMO specific protease 2 (SENP2), suggesting that this compound is a relatively nonselective isopeptidase inhibitor [94]. Later, using a unique quenched pair assay, compound 14 was identified as a reversible inhibitor of USP2 with an uncompetitive mechanism and an IC_50_ of 250 nM. It is also worth mentioning that the introduction of a fluorine atom reversed the selectivity between USP2 and USP7.

#### 5.2.6. USP4/USP5 Inhibitors

**Figure 11:**
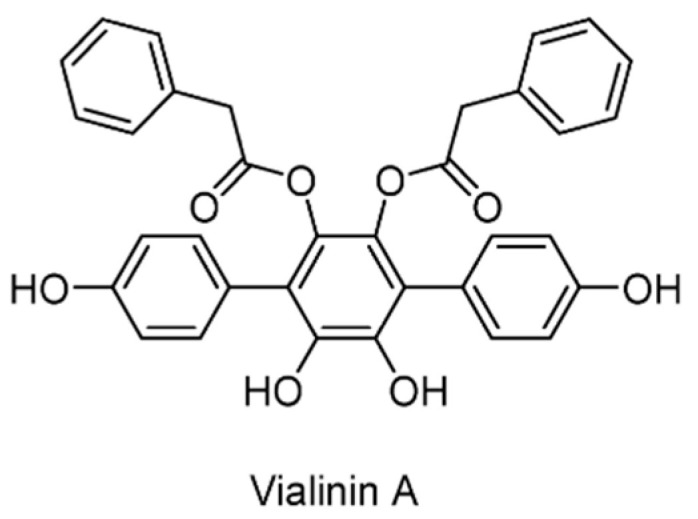
Structure of vialinin A.

Vialinin A (Figure 11), which was isolated from the Chinese mushroom Thelephora vialis, was shown to act as an inhibitor of USP5 (IC_50_ = 5.9 μM) and USP4 (IC_50_ = 1.5 μM) and possess significant anti-inflammatory activity [126]. However, no significant inhibition was observed for USP2, USP8, and UCHL3, which suggested that vialinin A was a semi-selective inhibitor. It led to a reduction in the ubiquitylation level of the inhibitor of NF-κB (I-κB) and the release of tumor necrosis factor-α (TNF-α).

#### 5.2.7. USP5/7/8/13/14/15/22 Inhibitors

**Figure 12:**
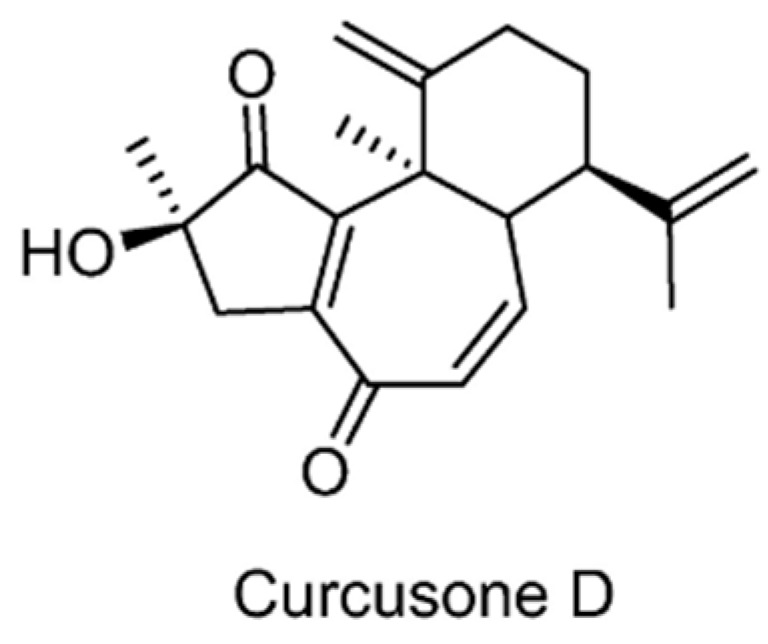
Structure of curcusone D.

Inspired by the discovery that the α,β-unsaturated carbonyl in prostaglandins could react with the catalytic cysteine of DUBs to form covalent bonds and result in the inhibition of the DUBs, curcusone D (Figure 12) was reported as a novel USPs inhibitor [127]. Curcusone D is a diterpene isolated from Jatropha curcas (Barbados nut), a herbal plant that has been used in traditional folk medicine in many tropical countries. As a non-selective DUBs inhibitor, curcusone D was shown to inhibit USP5, USP7, USP8, USP13, USP14, USP15, and USP22, but had no effect on UCHL1, UCHL3, and UCHL5. It also showed efficacy in multiple myeloma when used in combination with bortezomib.

#### 5.2.8. USP5/9X/14/24/UCHL5 Inhibitors

**Figure 13:**
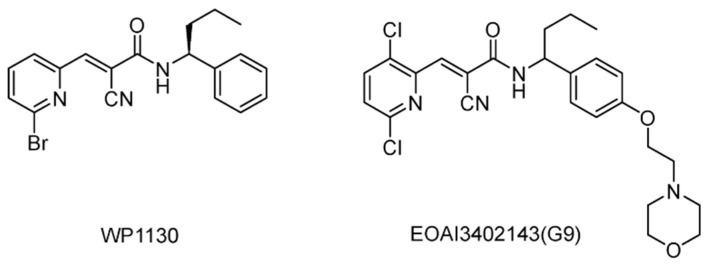
Structures of reported USP5/9X/14/24/UCHL5 inhibitors.

WP1130 (Degrasny) (Figure 13), which was initially identified as a JAKs/STATs pathway inhibitor, was later shown to directly inhibit USP9X, USP5, USP14, and UCHL5 [128,129]. As a cell-permeable DUBs inhibitor, WP1130 caused rapid accumulation of Lys48/Lys63 poly-ubiquitylated proteins, which led to the formation of near-nuclear aggresomes and eventually apoptosis without affecting 20S proteasome activity. Another study demonstrated that combined treatment with WP1130 sensitized hepatocellular carcinoma (HCC) cells to doxorubicin via USP9X-depedent p53 degradation [130]. Furthermore, it was later shown that WP1130-mediated USP9X inhibition prevents the growth of ERG-positive prostate tumors in vitro and in a mouse xenograft model [131].

EOAI3402143 (G9) (Figure 13) was identified in the study of WP1130 analogs, and showed improved drug-like properties and potency against USP9X [132]. Furthermore, G9 displayed USP24 inhibitory activity and potent apoptotic activity against myeloma and diffuse large B-cell lymphomas. A mechanism of action study indicated that G9 inhibits USP9X through a covalent and slow reversible conjugation with the cysteine residue.

#### 5.2.9. USP7 Inhibitors

**Figure 14:**
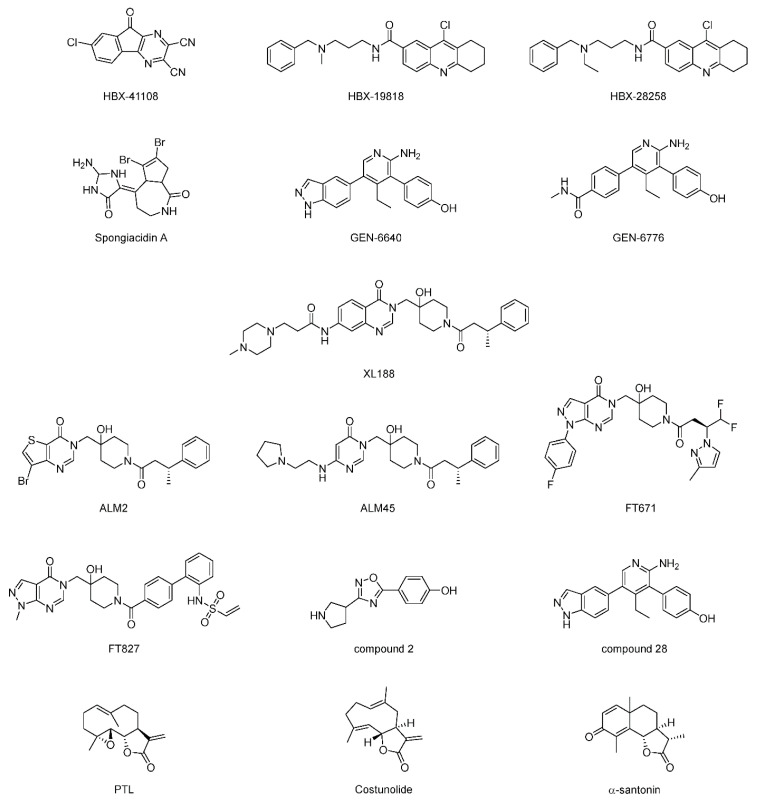
Structures of representative USP7 inhibitors.

Through high-throughput screening of a proprietary library of 65,092 chemically diverse compounds, HBX-41108 (Figure 14) was identified as a USP7 inhibitor (IC_50_ = 0.424 μM) [133]. It showed an uncompetitive reversible inhibition according to kinetics assay. It was shown to affect USP7-mediated p53 deubiquitylation in vitro and in cells. Later, HBX-19818 and HBX-28258 were also identified as USP7 inhibitors [134]. They were shown to be covalent inhibitors binding to the active site, and exhibited no cross-reactivity on other USP members including USP2, USP5, USP8, and USP20.

Spongiacidin A, isolated from a marine sponge, was identified as the first USP7 inhibitor (IC_50_ = 3.8 μM) from a natural source [135]. It exhibited the inhibition of USP21c (IC_50_ = 16.6 μM) as well. However, it did not show any cytotoxic activity.

**Figure 15:**
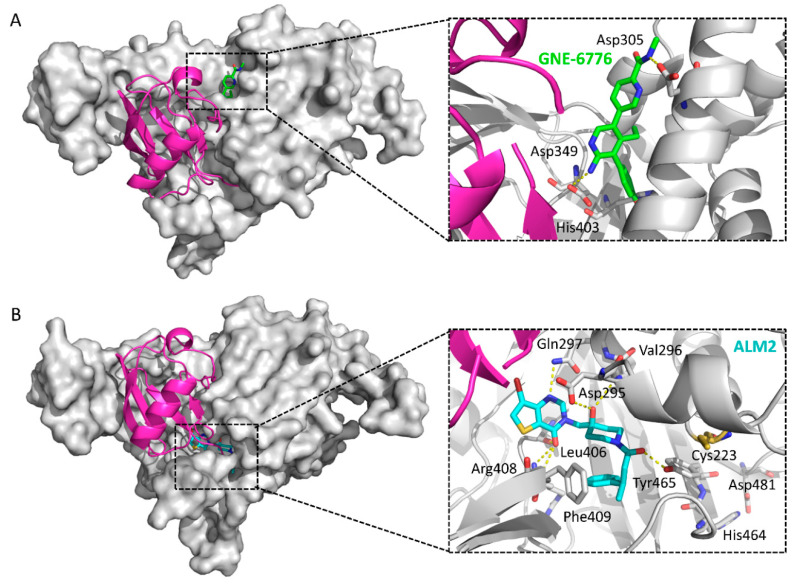
Comparison of the structures of USP7-GNE-6776 (PDB ID: 5UQX) and USP7-ALM2 (PDB ID: 5N9R). A ubiquitin molecule is modeled into the complexes based on a superposition with a USP7CD-ubiquitin complex (PDB ID: 1NBF). Ubiquitin: magenta; USP7 catalytic domain: gray; GNE-6776: green sticks; ALM2: cyan sticks. Hydrogen bonds: yellow dotted lines. Key residues involved in binding: sticks. (**A**) Crystal structure of USP7-GNE-6776 and a close-up of the GNE-6776-binding site. In the close-up figure, the ubiquitin peptide AGKQLED is omitted for clarity. (**B**) Crystal structure of USP7-ALM2 and a close-up of the ALM2-binding site. In the close-up figure, the catalytic triad of USP7 (Cys223-His464-Asp481) is also shown as sticks, with Cys223 in orange, and the ubiquitin C-terminal peptide LRLRGG is omitted for clarity.

An extensive screening, including HTS and NMR fragment screening followed by counter-screening against USP5 and USP47, was carried out, and a series of fragments with favorable properties was identified [136]. The optimization of these hit fragments ultimately yielded GNE-6640 and GNE-6776 (Figure 14), which showed considerable inhibitory activity against both full length (IC_50_ = 0.75 and 1.34 μM) and the USP7 catalytic domain (IC_50_ = 0.43 and 0.61 μM) [136]. Surprisingly, co-crystal structures revealed that this scaffold binds to a hydrophobic pocket approximately 12 Å away from the catalytic triad (PDB ID: 5UQV and 5UQX), indicating a new mode of inhibition rather than competing with the ubiquitin C-terminus (Figure 15A). By comparing with the ubiquitin-bound structure, GNE-6640 and GNE-6776 appeared to exert their inhibitory activity by sterically hindering ubiquitin binding and preventing the transition of USP7 α5 helix to the active conformation. Moreover, it is notable that GNE-6776 is orally bioavailable and promotes on-target pathway modulation.

A potent and selective inhibitor of USP7, XL188, with an IC_50_ of 90 nM, was developed by structure-based design [137]. XL188 specifically binds to the S4-S5 pocket of USP7 (PDB ID: 5VS6), indicating that XL188 is a non-covalent active-site inhibitor. Further studies have shown that XL188 caused the elevation of tumor suppressor proteins p53 and p21, thereby inhibiting the occurrence and development of tumors.

Fragment-based screening using surface plasmon resonance (SPR) on 1946 fragments against the catalytic domain of USP7 was carried out. By combining the fragment hits with known features of published USP7 inhibitors, followed by crystallography and rational structural modification, the authors achieved a highly potent inhibitor ALM2. It consistently exhibited IC_50_ values in the single-digit nanomolar range (IC_50_ = 6 nM, fluorescence polarization (FP) assay; IC_50_ = 1.5 nM, Ub-Rho110 assay) and demonstrated high selectivity (>10,000-fold) against other members of the USP and DUB families [138]. It is a non-competitive inhibitor. Furthermore, its antiproliferative effects were explored, and cell lines hypersensitive to USP7 inhibition (EC_50_ < 30 nM), including both haematological (RS4; 11) and solid tumor cell lines (LNCaP), were identified. The crystal structure of the USP7-ALM2 complex (PDB ID: 5N9R) shows that ALM2 occupies the ubiquitin C-terminal tail-binding channel, thus creating a steric clash with the ubiquitin C-terminus (Figure 15B). Key interactions include three hydrogen bonds contributed by the core 4-hydroxypiperadine, four hydrogen bonds contributed by the *3H*-pyrimidin-*4*-one group, and hydrophobic interactions with the side chain of Phe409. Notably, the stereochemistry of the methyl group in ALM2 is later confirmed to greatly affect the potency of the compounds, with the favored conformation of this scaffold being the (*R*)-stereoiomer.

By structure-guided design high-resolution crystallography, ALM45 was developed as a selective USP7 inhibitor with an IC_50_ of 0.1 μM (PDB ID: 6F5H) [139]. In particular, ALM45 demonstrated excellent biochemical and the absorption, distribution, metabolism, and excretion (ADME) profile, as well as promising pharmacokinetic profile.

A class of pyrazolo[3,4-d]pyrimidin-4-one-piperidine (PyrzPPip) compounds was identified through screening a diverse collection of approximately 500,000 compounds using a ubiquitin-Rho110 assay [140]. Further structural optimization of this series led to a non-covalent inhibitor, FT671 (IC_50_ = 52 nM), and a covalent inhibitor, FT827 (kincat/Ki= 66 M-1S-1) [141]. The cocrystal structures revealed that these two inhibitors target a dynamic pocket near the catalytic center of the auto-inhibited apo form of USP7, which differs from other USP deubiquitinases (PDB ID: 5NGE and 5NGF). Moreover, it was shown that FT671 destabilizes USP7 substrates including MDM2, upregulates p53 level, and results in the transcription of the target genes of p53. It also showed efficacy in the MM.1S xenograft mouse model.

Through integrated NMR and in silico techniques, two series of inhibitors were identified [142]. First, an oxadiazole series was identified by ligand-based virtual screening on an internal library and was represented by compound 2. Further biophysical characterization, including two-dimensional [^1^H-^15^N] TROSY spectrum and crystallization, revealed that compound 2 binds to a novel site within the “palm” regions (PDB ID: 5WHC). Second, an aminopyridine series was identified via an NMR-driven scaffold-hopping strategy. As a representative, compound 28 demonstrated submicromolar activity for USP7 (IC_50_ = 0.75 μM) and MDM2 expression (EC_50_ = 0.3 μM).

In a recent study in 2020, sesquiterpene lactone parthenolide (PTL) was reported as an inhibitor of USP7 (IC_50_ = 6.58 μM, Ub-AMC assay; IC_50_ = 15.42 μM, Ub-Rho110 assay) [143]. Treatment with PTL partially destabilized β-catenin, thereby inhibiting the activity of the Wnt pathway. Cytostatic experiments demonstrated that PTL prevented the proliferation of colorectal cancer cells and induced apoptosis. Two more sesquiterpene lactones (costunolide and α-santonin) were also identified to be USP7 inhibitors, indicating that the α-methylene-γ-butyrolactone can serve as a new scaffold for future development of USP7 inhibitors.

#### 5.2.10. USP7/8 Inhibitors

**Figure 16:**
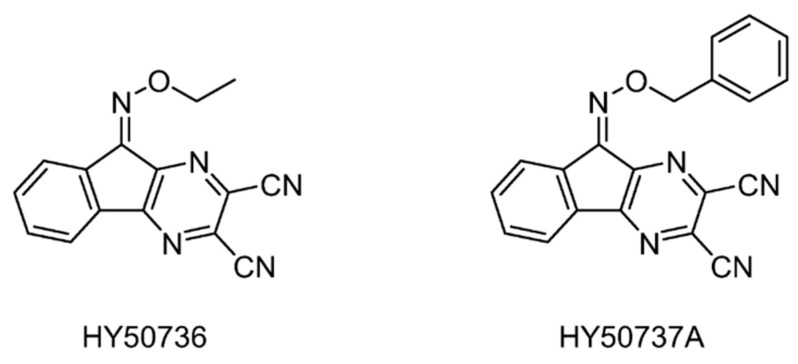
Structures of reported USP7/8 inhibitors.

On the basis of the USP7 inhibitor HBX-41108, USP7/8 activity was explored by structural modification [144]. In particular, the introduction of O-alkyloxime moieties at C-9 of the tricyclic scaffold gave the first known USP8-specific inhibitors with an IC_50_ below 1 μM, which was exemplified by HY50536 and HY50737A (IC_50_ = 0.28 and 0.24 μM) (Figure 16).

#### 5.2.11. USP7/10 Inhibitors

**Figure 17:**
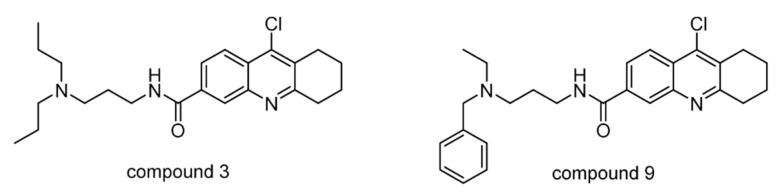
Structures of reported USP7/10 inhibitors.

A small series of HBX19818 analogs was evaluated for their inhibitory activity on USP10 [145]. Among them, compound 9 (Figure 17) inhibited USP10 similarly to HBX19818, but with no inhibition against USP7 (IC_50_ >> 100 μM). Compound 3 showed a lower anti-proliferation EC_50_ and induced FMS-like tyrosine kinase 3 (FLT3) degradation at lower concentrations.

#### 5.2.12. USP7/47 Inhibitors

**Figure 18:**
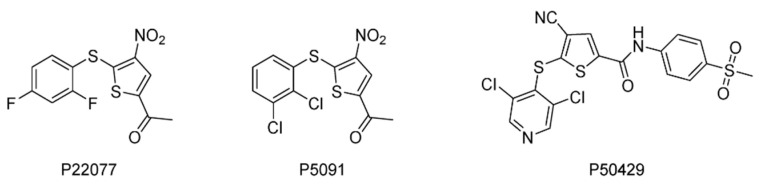
Structures of reported USP7/47 inhibitors.

The thiophenyl compound P22077 (Figure 18) was found to be a selective inhibitor of USP7 (IC_50_ = 8.0 μM) and induce cell death in HCT116 and HEK293T cells [122]. It was later shown to play a role in the treatment of non-small cell lung cancer [146]. It was also revealed that P22077 mediated the increase in intracellular reactive oxygen species by enhancing intracellular oxidative stress response and endoplasmic reticulum stress response, thereby causing apoptosis [147].

Through a high throughput screening, the trisubstituted thiophenyl compound P5091 was discovered to be a USP7 inhibitor (IC_50_ = 4.2 μM) [148]. It showed selectivity toward USP7, with no effect on other DUBs such as USP2 and USP8. P5091 can induce the apoptosis of multiple myeloma (MM) cells resistant to traditional therapy or bortezomib by inhibiting the activity of USP7, and can also play a synergistic role when used in combination with dexamethasone or lenalidomide. Furthermore, it exhibited no cytotoxicity in the USP7 knockout HCT116 cell line, indicating that the cytotoxicity of P5091 depends on the intracellular USP7. It can induce cell death in ovarian cancers with different p53 status [149]. It also suppressed in vivo tumor growth in the HCT116 xenograft mouse model, which is consistently associated with reduced expression of β-catenin and Wnt target genes [150].

Progenra conducted the structural modification of P5091 and demonstrated that after the acetyl group in the C-2 position was changed to various amides, the derivative P50429 showed an IC_50_ of 0.42 μM and 1.0 μM against USP7 and USP47, respectively [151]. It has no inhibitory effect on apoptosis-related caspase 1/3 or 20S proteasome. In addition, the IC_50_ values of P50429 for other USP family members such as USP2, USP5, USP8, USP21, and USP28 are all greater than 31.6 μM.

#### 5.2.13. USP10/13 Inhibitors

**Figure 19:**
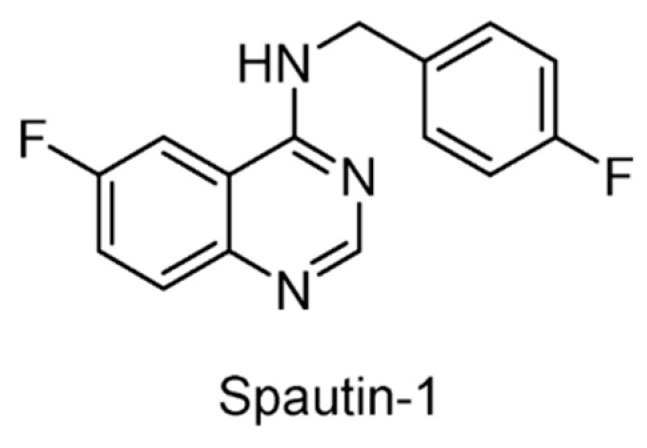
Structure of Spautin-1.

Through an imaging-based screening and subsequent structural modification, a potent autophagy inhibitor spautin-1 (Figure 19) was identified, which was demonstrated to inhibit USP10 and USP13 and thus promote the degradation of Vps34 PI3 kinase complexes [152].

#### 5.2.14. USP11/15 Inhibitors

**Figure 20:**
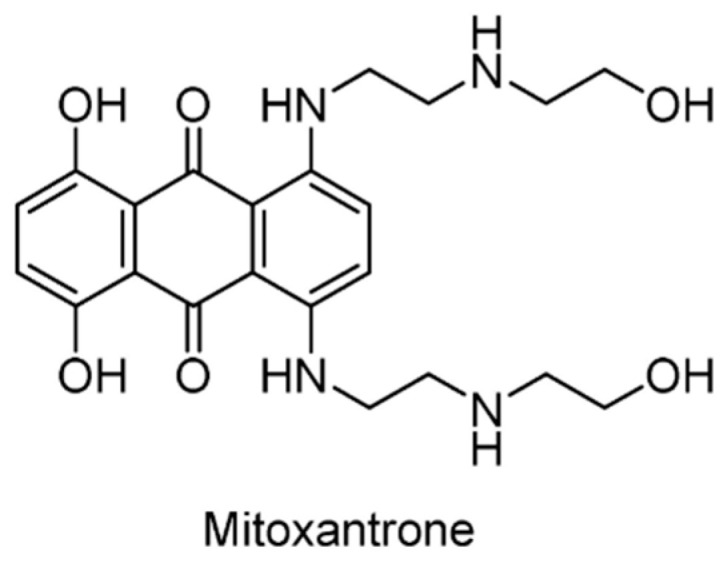
Structure of mitoxantrone.

**Figure 21:**
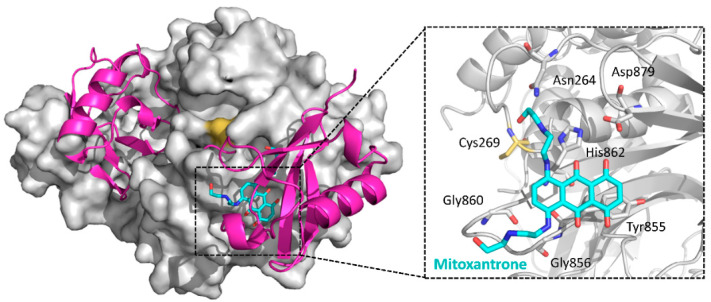
Crystal structure of the USP15-mitoxantrone complex and a close-up of the binding site (PDB ID: 6GH9). A di-ubiquitin molecule is modeled into the complex based on a superposition with a USP30 C77A Lys^6^-linked di-ubiquitin structure (PDB ID: 5OHP), the closest available USP structure in complex with a substrate. Ubiquitin: magenta; USP15 catalytic domain: gray; mitoxantrone: cyan sticks. In the close-up figure, key residues involved in binding and the catalytic triad (Cys269-His862-Asp879) are shown as sticks, with Cys269 marked orange, and the di-ubiquitin molecule is omitted for clarity.

Mitoxantrone (Figure 20), a clinical drug used to treat acute myeloid leukemia, hormone refractory prostate cancer, and multiple sclerosis treatment, was reported to inhibit USP11 (IC_50_ = 3.15 μM) and impact pancreatic ductal adenocarcinoma (PDA) cell survival [153]. In addition, it was found that mitoxantrone weakly inhibits the activity of USP15 with an IC_50_ of 33 µM. The crystal structure of the USP15-mitoxantrone complex (PDB ID: 6GH9) revealed predominantly hydrophobic interactions between mitoxantrone and USP15 residues Tyr855, Gly856, Gly860 and His862, which are located near the catalytic Cys269 (Figure 21) [154].

#### 5.2.15. USP14 Inhibitors

**Figure 22:**
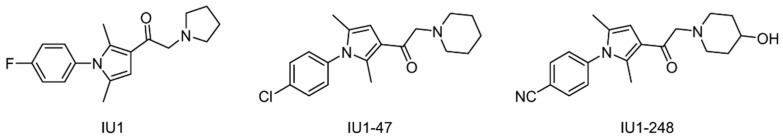
Structures of representative USP14 inhibitors.

By screening 63,052 compounds, IU1 (Figure 22) was identified as the first USP14 inhibitor (IC_50_ = 4 μM) [155]. It interacts with the active form of USP14, blocking its docking to proteasome. It had little or no inhibitory activity against eight other DUBs: isopeptidase T (IsoT/USP5), UCHL5, BAP1, UCHL1, UCHL3, USP15, USP2, and USP7. IU1 promoted the degradation of several proteasome substrates that have been implicated in neurodegenerative disease, indicating that IU1 may play an important role in the drug development for neurodegenerative disease.

Later, during the structural modification of IU1, IU1-47 was identified as a potent USP14 inhibitor with an IC_50_ of 0.6 μM, accompanied by a modest increase in selectivity over IsoT/USP5 to approximately 33-fold [156]. Moreover, using the microtubule-associated protein tau that has been implicated in many neurodegenerative diseases as a reporter, IU1-47 was shown to enhance protein degradation in cells.

**Figure 23:**
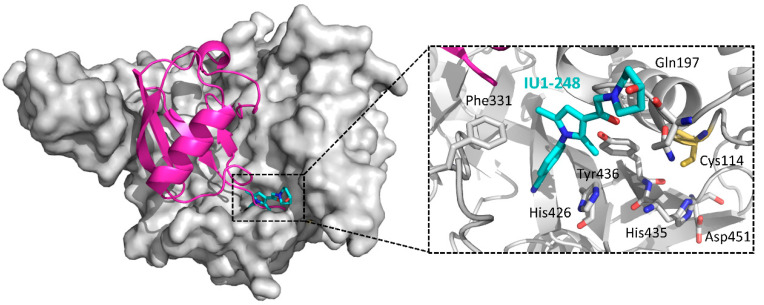
Cocrystal structure of USP14-IU1-248 and a close-up of the binding site (PDB ID: 6IIN). A ubiquitin molecule is modeled into the complex based on the superposition with a USP14CD-ubiquitin complex (PDB ID: 2AYO). Ubiquitin: magenta; USP14 catalytic domain: gray; IU1-248: cyan sticks. Hydrogen bonds: yellow dotted lines. Key residues involved in binding and the catalytic triad (Cys114-His435-Asp451) are shown as sticks, with Cys114 in orange. The ubiquitin C-terminal peptide RLRGG is omitted for clarity.

The high-resolution co-crystal structures of USP14 (PDB ID: IU1, 6IIK; IU1-47, 6IIL) revealed that IU1 and its analogs bind competitively with the C-terminus of ubiquitin to the active site of USP14, thereby abrogating the catalytical activity of USP14 [157]. Subsequent structure-guided design led to the discovery of IU1-248 with an IC_50_ of 0.83 μM. Taking the USP14-IU1-248 cocrystal structure as an example, as shown in Figure 23, the phenyl ring of the inhibitor extends into the inner hydrophobic pocket constituting of Phe331, His246, and Tyr436, while the piperidine ring provides both hydrogen bond and hydrophobic interactions.

#### 5.2.16. USP14/UCLH5 Inhibitors

**Figure 24:**
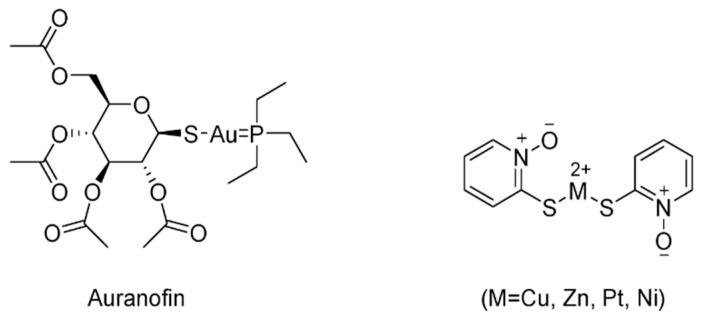
Structures of representative USP14/UCLH5 inhibitors.

In recent years, several metal-based compounds have been found to target USP14 with promising therapeutic value [158]. One of the representative drugs is auranofin (Aur) (Figure 24), a gold-containing compound, which has been used clinically to treat rheumatic arthritis since 1985. Aur recently entered phase 2 clinical trials as a cancer therapy. Later, it was demonstrated that Aur targets both UCHL5 and USP14 [159]. Moreover, the in vivo efficacy of Aur was evaluated in mouse xenograft models, suggesting that Aur can accumulate proteasome substrates and inhibit tumor growth.

In addition, various pyrithione (PT)-metal chelates, including copper, zinc, nickel, and platinum, have been reported as USP14/UCHL5 inhibitors [160,161,162,163]. ZnPT, an FDA-approved drug, was reported to target USP14 and UCHL5, as well as efficiently inducing apoptosis in primary cancer cells from leukemia patients and suppressing tumor growth in mouse xenografts [161]. Platinum pyrithione (PtPT) and nickel pyrithione (NiPT) were also successfully explored as inhibitors inducing typical proteasome inhibition via targeting USP14/UCHL5 [162,163]. CuPT has also been reported as a novel class of USP14/UCHL5 inhibitors [160].

#### 5.2.17. USP25/28 Inhibitors

**Figure 25:**
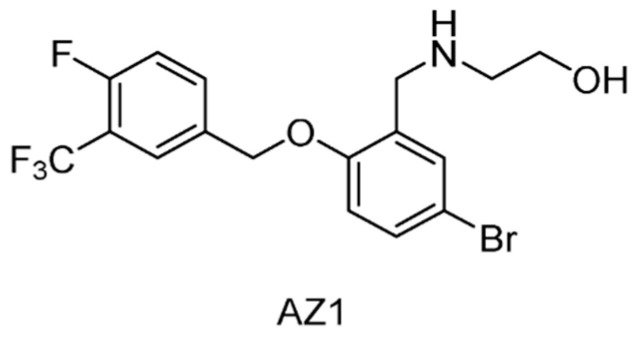
Structure of compound AZ1.

By high-throughput screening on a directed library of approximately 40,000 compounds, AZ1 (Figure 25) was identified as the first known USP28 inhibitor exhibiting an IC_50_ of 0.7 μM [164]. Interestingly, subsequent selectivity profiling against USPs and DUBs demonstrated that AZ1 had strong inhibition against USP25 (IC_50_ = 0.62 μM), whilst no significant effect was observed against any of the other family members tested (<10% inhibition at 10 μM). Furthermore, it was shown that AZ1 was responsible for the modulation of both the levels and the half-life of c-Myc, and apoptosis and loss of cell viability in a range of cancer cell lines.

#### 5.2.18. USP30 Inhibitors

**Figure 26:**
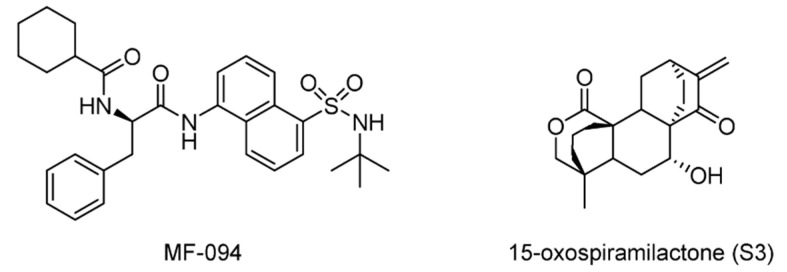
Structures of reported USP30 inhibitors.

During a hit-to-lead optimization, MF-094 (Figure 26) was identified as a potent and selective USP30 inhibitor (IC_50_ = 0.12 μM) [165]. It was shown that MF-094 accelerated the disappearance of 5-bromo-2′-dexoyuridine (BrdU) from the mitochondrial DNA, indicating the role of MF-094 in the acceleration of mitophagy.

In a study to understand mitochondrial fusion and fission, a diterpenoid 15-oxospiramilactone (S3) was found to induce remarkable mitochondrial elongation in cells that lack mitofusin 1 (Mfn1). Later, a mechanism of action study determined that S3 directly interacted with USP30, suggesting that USP30 is a target of S3 [166].

## 6. Conclusions

Ubiquitylation and deubiquitylation control nearly all aspects of human cell biology and physiology, while any defects can cause diseases. Accordingly, ubiquitylation and related processes have drawn tremendous attention as potential therapeutic targets. USPs, as the most widely studied enzymes in the deubiquitinating enzyme family, have been proposed as promising targets for anticancer, antiviral, and anti-infective treatments. During the past decades, in-depth studies on USPs in human cancer progression have achieved significant advances such as in the identification of their pathological functions, mode of actions, intrinsic molecular mechanisms, and regulation in cancer. Several USPs are responsible for tumorigenesis by various cancer-related signaling pathways, including the DNA repair pathway, TGF-β pathway, p53 pathway, etc., whereas one USP can generally affect more than one pathway. All these findings contributed to the development of small molecule inhibitors against USPs with anticancer potency, which could, in turn, help to explore or/and confirm the precise functions of the targeted USPs. For example, USP7 has been discovered to stabilize both MDM2 and p53 by deubiquitylation, with a higher binding affinity for MDM2 according to structural and biochemical data [32,33]. In another work, HBX-41108, a USP7-specific inhibitor, increases p53 levels in HCT116 colon cancer cells by inhibiting MDM2 deubiquitylation [133].

Considering the fact that proteolysis-targeting chimeras (PROTACs), perhaps one of the most exciting technologies to arise in recent years, implement aimed protein degradation based on the UPS system, it is worthy to briefly compare USP inhibitors with PROTACs. These two strategies do differ greatly and have totally distinct advantages and disadvantages. The PROTACs technology employs E3 ligase ligands via a flexible chemical linker to the target protein to elicit ectopic ubiquitylation, thus promoting the UPS-mediated degradation of a specific target protein, whereas USP inhibitors induce targeted degradation through the inhibition of deubiquitylation process. Since PROTACs are still an emerging technology, though their small molecule nature makes PROTACs simple to use in experiments as inhibitors, the conversion of an E3 ligase ligand into a PROTAC can still be time-consuming, and, of course, not all proteins or subcellular locations are amenable yet. USP inhibitors, however, do not have these hindrances but generally suffer from a lack of selectivity because members of USPs share a high degree of homology.

The discovery of USP inhibitors reported before 2014 mainly relied on high-throughput screening. These studies rarely included any structure-activity relationship (SAR), lacking compound optimization and rigorous characterization. Recently, only after the co-crystal structures of USP-inhibitor complexes were reported, it became feasible to take advantage of structure-guided drug design and carry out SAR optimization. Interestingly, several inhibitors have been shown to bind to the vicinity of the catalytic site instead of the central region where the catalytic cysteine is located. Meanwhile, biophysical and biochemical assays including SPR, isothermal titration calorimetry (ITC), differential scanning fluorimetry (DSF) and hydrogen-deuterium exchange mass spectrometry (HDX-MS) started to be used for on-target binding validation. Current efforts are mainly focused on the improvement of the compound inhibitory selectivity against the entire USPs or DUBs family. With the continued improvements in selectivity profile, target engagement, and phenotypic discovery, we will expand our understanding of the exact action mode of USP inhibitors and their selectivity. More potent and selective USP inhibitors or even clinical candidates will be discovered and eventually developed into useful therapeutic agents.

## Figures and Tables

**Table 1:** Cancer-related pathways regulated by USPs.

Pathway	USPs Involved	Refs
DNA damage repair	USP1, USP28	[35,36]
TGF-β	USP2a, USP4, USP9X, USP15, USP26	[34,37,38,39,40]
Wnt/β-catenin	USP4, USP5, USP9X, USP14	[41,42,43,44,45]
p53	USP2, USP4, USP5, USP7, USP10, USP15, USP24, USP42	[31,32,33,46,47,48,49,50,51,52,53,54,55]
c-Myc	USP2, USP10, USP22, USP28, USP36, USP37	[56,57,58,59,60,61]
Akt	USP4, USP12, USP14, USP22, USP46	[62,63,64,65,66,67]
JAKs-STATs	USP7	[68]
NF-κB	USP4, USP11, USP14, USP15, USP18, USP19, USP20, USP35, USP24, USP48	[69,70,71,72,73,74,75,76,77,78,79,80,81,82]
GPCR	USP4, USP8, USP14, USP20, USP30	[83,84,85,86]

**Table 2:** List of reported USP inhibitors.

Target	Compound ID	PDB	Refs
USP inhibitors in clinical trials			
USP14/UCHL5	b-AP15	None	[103]
	VLX1570	None	[109]
USP inhibitors of reported new chemical entities			
USP1	Pimozide	None	[113]
	GW7647	None	
	Trifluoperazine	None	
	Rottlerin	None	
	C527	None	[114]
	SJB2-043	None	
	SJB3-091A	None	
	ML323	None	[115,116]
USP2	Q29	None	[117]
	ML364	None	[118]
	LCAHA	None	[119]
	STD1T	None	[120]
	6TG	5XU8	[121]
USP2/4/5/7/8/15/20/28/47/UCHL1/UCHL3/UCHL5	PR619	None	[122,123]
USP2/5/8/UCHL1/UCHL3	AM416	None	[124]
	RA9	None	
	RA14	None	
USP2/7	NSC632839	None	[98]
	Compound 14	None	[125]
USP4/5	Vialinin A	None	[126]
USP5/7/8/13/14/15/22	Curcusone D	None	[127]
USP5/9X/14/24/UCHL5	WP1130 (Degrasny)	None	[128,129,130,131]
	EOAI3402143 (G9)	None	[132]
USP7	HBX-41108	None	[133]
	HBX-19818	None	[134]
	HBX-28258	None	
	Spongiacidin A	None	[135]
	GEN-6640	5UQV	[136]
	GEN-6776	5UQX	
	XL188	5V6S	[137]
	ALM2	5N9R	[138]
	ALM45	6F5H	[139]
	FT671	5NGE	[140,141]
	FT827	5NGF	
	Compound 2	5WHC	[142]
	Compound 28	None	
	Parthenolide (PTL)	None	[143]
	Costunolide	None	
	α-santonin	None	
USP7/8	HY50736	None	[144]
	HY50737A	None	
USP7/10	Compound 3	None	[145]
	Compound 9	None	
USP7/47	P22077	None	[122,146,147]
	P5091	None	[148,149,150]
	P50429	None	[151]
USP10/13	Spautin-1	None	[152]
USP11/15	Mitoxantrone	6GH9	[153,154]
USP14	IU1	6IIK	[155]
	IU1-47	6IIL	[156,157]
	IU1-206	6IIM	[157]
	IU1-248	6IIN	[157]
USP14/UCHL5	Auranofin	None	[158,159]
	CuPT	None	[160,161]
	PtPT	None	[162]
	NiPT	None	[163]
USP25/28	AZ1	None	[164]
USP30	MF-094	None	[165]
	15-oxospiramilactone (S3)	None	[166]

## Data Availability

Not applicable. No new data were created or analyzed in this study. Data sharing is not applicable to this article.
